# ﻿Emended Neodactylariales (Dothideomycetes): *Szaferohypha* gen. nov. and phylogenetically related genera

**DOI:** 10.3897/mycokeys.111.139620

**Published:** 2024-12-27

**Authors:** Marcin Piątek, Monika Stryjak-Bogacka, Paweł Czachura

**Affiliations:** 1 W. Szafer Institute of Botany, Polish Academy of Sciences, Lubicz 46, PL-31-512 Kraków, Poland Polish Academy of Sciences Kraków Poland

**Keywords:** Ascomycota, emended descriptions, one new genus, one new species, sooty mould communities, taxonomy

## Abstract

Epiphytic fungi evolved several times in Dothideomycetes, particularly within the orders Asterinales, Capnodiales, Microthyriales, and Zeloasperisporiales, but also in other, less obvious lineages. In this study, a new genus and species, *Szaferohypha* and *S.enigmatica*, isolated from the sooty mould community on the leaves of *Symphoricarposalbus* in Poland, are described based on morphology and phylogenetic analysis using sequences of four DNA loci (LSU, ITS, SSU, and *rpb2*). Due to single isolation, it is unclear whether *Szaferohyphaenigmatica* represents a very rare or accidental inhabitant of sooty mould communities. *Szaferohypha* is assigned to the poorly known family Neodactylariaceae and order Neodactylariales, together with *Beaucarneamyces*, *Neodactylaria*, and *Pseudoarthrographis*. The order and family were originally circumscribed based on the features of the genus *Neodactylaria*. Therefore, they are emended by characters of *Beaucarneamyces*, *Pseudoarthrographis*, and *Szaferohypha*.

## ﻿Introduction

The class Dothideomycetes is megadiverse and the largest class of fungi in the phylum Ascomycota (Wijayawardene et al. 2014, 2020). Currently, the class is divided into 54 orders and 226 families and contains about 20,000 described species (Abdollahzadeh et al. 2020; Hongsanan et al. 2020a, 2020b; Barreto et al. 2024; Pem et al. 2024; Piątek et al. 2024). The best-known and studied orders are Capnodiales s. lato, Dothideales, Botryosphaeriales, and Pleosporales (Zhang et al. 2012; Phillips et al. 2013, 2019; Quaedvlieg et al. 2014; Thambugala et al. 2014; Yang et al. 2017; Videira et al. 2017; Abdollahzadeh et al. 2020). Other orders of Dothideomycetes are less known, primarily because their members are less common in the environment, although some of them are important for human life and the economy (Hongsanan et al. 2020b; Shen et al. 2020).

The high species diversity observed in Dothideomycetes is reflected by the wide geographical range and diversity of lifestyles of its members. They are known from all areas of the world and habitats, even capable of colonizing extreme habitats such as cold deserts (Coleine et al. 2020), deep-sea sediments, including methane sediments (Nagahama et al. 2011; Rojas-Jimenez et al. 2020), saline waters (Czachura et al. 2021), acidic environments (Kolařík et al. 2021), and resin exudates (Czachura and Janik 2024). Species of Dothideomycetes are plant pathogens, saprobes, rock-inhabiting fungi, lichens, endophytes, and epiphytes. Hongsanan et al. (2016) defined fungal epiphytes as species occurring on the living plant surfaces, especially leaves, which belong to the following orders of Dothideomycetes: Asterinales, Capnodiales, Microthyriales, Zeloasperisporiales, and Meliolales in Sordariomycetes. However, epiphytes are also known in other, less obvious lineages (Piątek et al. 2023, 2024). The special kind of epiphytes are sooty moulds, which live on leaves/needles covered with exudates of phloem-feeding insects, especially honeydew secreted by aphids (Hughes 1976).

Recently, we isolated an enigmatic fungus from a sooty mould colony on *Symphoricarposalbus* in southern Poland, which showed affinities to members of the poorly known order Neodactylariales (Qiao et al. 2020; Crous et al. 2024). In this study, a novel species accommodated in a new genus is described for this fungus. The phylogenetic placement of this new genus and its most closely related genera are analysed, and emended descriptions of the order Neodactylariales and family Neodactylariaceae are provided.

## ﻿Materials and methods

### ﻿Strains and morphological analyses

The strain was isolated from the sooty mould community on *Symphoricarposalbus* leaves planted in municipal greenery in southern Poland (see Piątek et al. 2023). Macroscopic features of cultures were observed and photographed using 4-week-old colonies grown on MEA and PDA at 6 °C, 15 °C, and 25 °C, as well as 15-week-old colonies grown on MEA and PDA at 15 °C. Description of culture characteristics is based on 4-week-old colonies. The morphology of colonies observed after 15 weeks is briefly mentioned in the subsection “notes”. Growth at different temperatures was assessed by measuring the colony diameter after 4 weeks. Microscopic features were analysed using colonies older than 8 weeks grown on MEA at 15 °C. Hyphae and conidia taken from the edge of the colony were mounted in lactic acid (80%) on microscope slides and examined under a Nikon Eclipse 80i light microscope. Microscopic structures were measured and photographed using NIS‐Elements BR 3.0 imaging software. The holotype is a dried specimen obtained from culture and deposited in the fungal collection of the W. Szafer Institute of Botany, Polish Academy of Sciences, Kraków (KRAM F). Culture is preserved in the culture collection of the Westerdijk Fungal Biodiversity Institute (CBS) and in the W. Szafer Institute of Botany, Polish Academy of Sciences, Kraków. The morphological characteristics of *Beaucarneamyces* and *Pseudoarthrographis* used for the emendation of Neodactylariales and Neodactylariaceae are taken from the literature (Crous et al. 2018, 2024).

### ﻿DNA isolation, PCR, and sequencing

After growth of cultures for about one month, genomic DNA was extracted from a portion of mycelium using the DNeasy® Plant Mini Kit (Qiagen, Germany). To conduct molecular studies, four genetic loci were amplified, namely ITS1-5.8S-ITS2 rDNA (= ITS), 28S D1–D2 rDNA (= LSU), a small subunit rDNA (= SSU), and a protein-coding gene—a partial DNA-directed RNA polymerase II second largest subunit (*rpb2*). To amplify the loci mentioned above, four different primer pairs were used: ITS1 and LR5 for a fragment containing ITS and LSU (White et al. 1990; Vilgalys and Hester 1990), NS1 and NS4 for SSU (White et al. 1990), and fRPB2-5F and fRPB2-7cR for *rpb2* (Liu et al. 1999). Polymerase chain reactions (PCR) for all loci were performed in a reaction mixture prepared as described in Piątek et al. (2023). Amplification conditions for PCR reactions of the fragment containing ITS and LSU were performed as described by Piątek et al. (2023); in turn, amplification conditions for SSU and *rpb2* were described by Piątek et al. (2024). The PCR products were checked on 1% agarose gels and enzymatically purified using Exo-BAP Mix (EURx, Gdańsk, Poland). DNA sequencing was carried out in both directions by Macrogen Europe B.V. (Amsterdam, the Netherlands). ITS was sequenced using primers ITS1 and ITS4; LSU was sequenced using primers LSU1Fd and LR5; and SSU and *rpb2* were sequenced with the same pairs of primers that were used for amplification.

### ﻿Phylogenetic analysis

The affinities of obtained ITS, LSU, SSU, and *rpb2* sequences of the isolated fungus were performed in the NCBI’s GenBank nucleotide database using the megablast search tool (Zhang et al. 2000). To resolve its phylogenetic position, the multilocus dataset containing LSU, ITS, SSU, and *rpb2* sequences of representatives of Dothideomycetes used by Maharachchikumbura et al. (2021, with some modifications) was obtained. The dataset was, among others, augmented by sequences of representatives of the orders Neodactylariales (*Beaucarneamyces*, *Neodactylaria*, *Pseudoarthrographis*) and Oncopodiellales (*Diplocladiella*, *Oncopodiella*) (Suppl. material 1). Sequences were separately aligned for each single-gene dataset using the MAFFT algorithm (Katoh et al. 2005) in Geneious 11.1.5 and concatenated. Phylogenetic relationships were inferred using the concatenated LSU-ITS-SSU-*rpb2* alignment by the maximum likelihood (ML) analysis using RAxML-NG v. 1.1.1 (Kozlov et al. 2019), with 1000 bootstrap replicates. The best-fit substitution models were selected with ModelTest-NG v. 0.2.0 using the Bayesian Information Criterion (BIC) (Darriba et al. 2020). The final phylogenetic tree was visualized using FigTree v1.4.3. The alignment was deposited at figshare.com (https://doi.org/10.6084/m9.figshare.27231756.v1).

## ﻿Results

### ﻿Phylogenetic analysis

**Figure 1.**
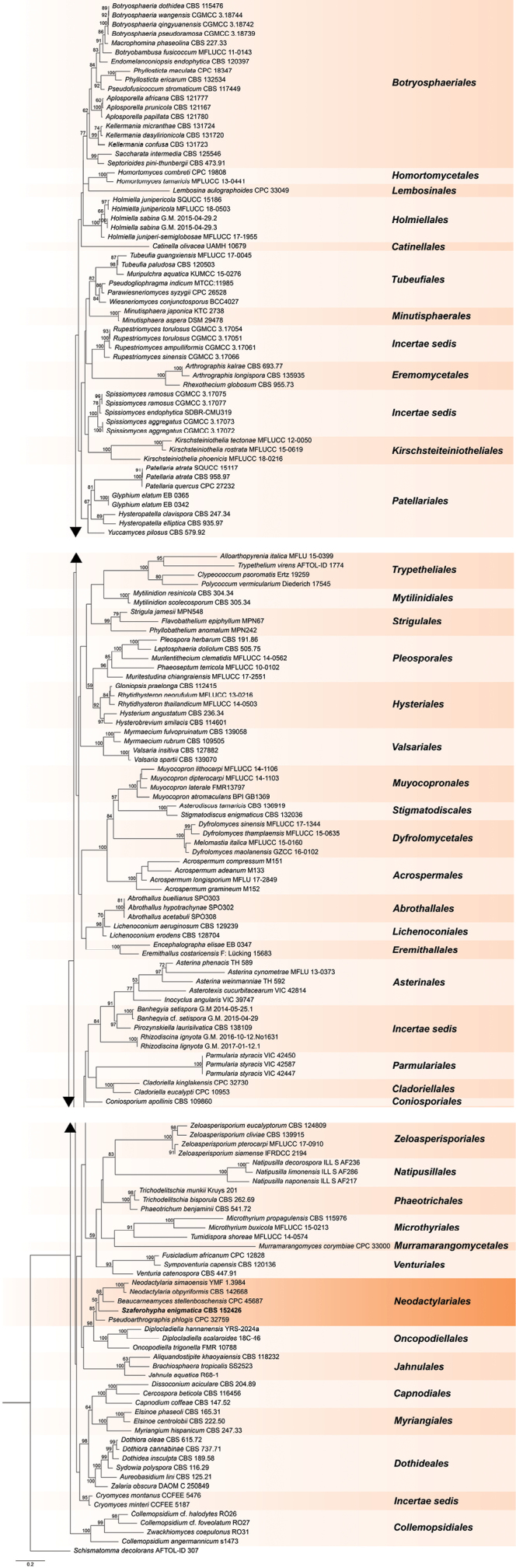
Phylogenetic tree of selected representatives of the orders of Dothideomycetes, including all sequenced species of Neodactylariales, obtained from a maximum likelihood analysis of the combined multi-locus alignment (LSU, ITS, SSU, *rpb2*). The position of *Szaferohyphaenigmatica* is indicated in bold. Numbers above branches indicate maximum likelihood bootstrap (MLB) support values > 50%. *Schismatommadecolorans* AFTOL-ID 307 was used as an outgroup. The scale bar represents the expected number of changes per site.

The concatenated multilocus dataset (LSU, ITS, SSU, and *rpb2*) included sequences of 154 representatives of most of the orders of the class Dothideomycetes, including all representatives of Neodactylariales, and a member of the class Arthoniomycetes (*Schismatommadecolorans*) used as an outgroup. The concatenated alignment contained 4483 characters (LSU: 1082, ITS: 750, SSU: 1761, *rpb2*: 890, including alignment gaps). The best-fit substitution models selected for single-gene alignments were as follows: GTR+I+G4 for both ITS and LSU, TrNef+I+G4 for SSU, and TPM3uf+I+G4, TPM2uf+I+G4, and TIM2+I+G4 for *rpb2* (three codons). The phylogenetic tree resulting from maximum likelihood analysis is shown in Fig. 1. Representatives of Dothideomycetes formed lineages that correspond to the orders that were well supported, but relationships between orders were mostly not resolved. The strain of a new genus and species, *Szaferohyphaenigmatica*, clustered within the lineage assigned to the order Neodactylariales, but its relationship to the remaining genera of this order (*Beaucarneamyces*, *Neodactylaria*, and *Pseudoarthrographis*) was not resolved. The clustering of genera assigned to Neodactylariales was well supported (MLB = 85%), and this order formed a strongly supported (MLB = 98%) sister group to the lineage assigned to the order Oncopodiellales that was fully supported (MLB = 100%).

### ﻿Taxonomy

#### ﻿ Neodactylariales

Taxon classificationFungiNeodactylarialesNeodactylariaceae

H. Zheng & Z.F. Yu, MycoKeys 73: 74 (2020), emend. Piątek, Stryjak-Bogacka & Czachura

B462339F-821C-5248-B4D8-13398D9CA787

##### Description.

Asexual morph from human-associated organs or saprobic on plant debris **or epiphytic on living leaves**. Conidiophores acroauxic, macronematous **or micronematous**, mononematous, branched or unbranched, **or reduced to conidiogenous cells**. Conidiogenous cells mono- and polyblastic, sympodially extended **or not**. Conidia solitary **or in branched or unbranched chains**, hyaline or pale pigmented, smooth, verrucous, or echinulate. **Chlamydospores sometimes present.** Sexual morph not observed.

##### Type genus.

*Neodactylaria* Guevara-Suarez, Deanna A. Sutton, Wiederh. & Gené.

##### Notes.

Because of the inclusion of genera *Beaucarneamyces*, *Pseudoarthrographis*, and *Szaferohypha* in Neodactylariales (Crous et al. 2024; this study), the emended description of this order is necessary. The original description of this order (Qiao et al. 2020) is included without changes and emended by crucial characters of these genera given in bold.

#### ﻿ Neodactylariaceae

Taxon classificationFungiNeodactylarialesNeodactylariaceae

H. Zheng & Z.F. Yu, MycoKeys 73: 75 (2020), emend. Piątek, Stryjak-Bogacka & Czachura

B6ABE117-6D66-58A5-B038-493B2ABD34E6

##### Description.

Mycelium superficial or immersed, composed of branched, septate, hyaline to subhyaline hyphae. Conidiophores macronematous **or micronematous**, mononematous, straight or flexuous, septate, unbranched, **or reduced to conidiogenous cells**. Conidiogenous cells terminal or intercalary, **monoblastic or** polyblastic, sympodial **or not**, with short-cylindrical denticles **or without denticles**. Conidial secession schizolytic. Conidia solitary **or in branched or unbranched chains**, smooth or finely echinulate. **Chlamydospores sometimes present.** Sexual morph not observed.

##### Type genus.

*Neodactylaria* Guevara-Suarez, Deanna A. Sutton, Wiederh. & Gené.

##### Notes.

The order Neodactylariales contains one family, Neodactylariaceae, that is emended by features of genera *Beaucarneamyces*, *Pseudoarthrographis*, and *Szaferohypha*. The original description of this family (Qiao et al. 2020) is included without changes, and the emended part is given in bold.

#### ﻿ Szaferohypha

Taxon classificationFungiNeodactylarialesNeodactylariaceae

Piątek, Stryjak-Bogacka & Czachura gen. nov.

3497AF02-D430-58CD-9593-E0740167931A

856722

##### Etymology.

Named after Polish botanist and palaeobotanist Professor Władysław Szafer (1886–1970), the first director of the W. Szafer Institute of Botany, Polish Academy of Sciences.

##### Description.

Colonies erumpent, spreading, umbonate, grayish-brown, with a velvety surface caused by abundant aerial mycelium, margin undulate. Mycelium composed of branched, septate, hyaline, subhyaline, pale brown, or brown, smooth or verrucose, usually thick-walled hyphae. Conidiophores micronematous, reduced to conidiogenous cells, rarely macronematous. Conidiogenous cells terminal, rarely lateral, monoblastic, hyaline, subhyaline, pale brown, or brown. Conidia globose, subglobose, rarely broadly ellipsoid, hyaline, subhyaline, or brown, aseptate, rarely with 1–2 septa or muriformly septate, smooth or finely verrucose, thick-walled, sometimes produced intercalary.

##### Type species.

*Szaferohyphaenigmatica* Piątek, Stryjak-Bogacka & Czachura.

#### ﻿ Szaferohypha enigmatica

Taxon classificationFungiNeodactylarialesNeodactylariaceae

Piątek, Stryjak-Bogacka & Czachura sp. nov.

48C7118F-FEEF-54F1-9AB3-E5553922377E

856723

Figs 2 , 3 , 4

##### Etymology.

Refers to the uncertain taxonomic position of this fungus after the first molecular analyses.

##### DNA barcodes.

ITS (PQ479987), LSU (PQ479989), SSU (PQ479988), *rpb2* (PQ475069).

##### Typus.

Poland • Małopolska Province, Tarnów County: Tarnów–Piaskówka, municipal greenery, isolated from sooty mould community on *Symphoricarposalbus* leaves, 1 Oct. 2018, leg. M. Piątek, W. Bartoszek & P. Czachura (holotype KRAM F-59996; culture ex-type: G191 = CBS 152426).

##### Description.

Mycelium composed of branched, septate, hyaline, subhyaline, pale brown, or brown, smooth or verrucose, usually thick-walled hyphae, 2–4 µm, sometimes with oil guttules; wall ca. 0.5 µm thick. Conidiophores micronematous, reduced to conidiogenous cells, rarely macronematous. Conidiogeneous cells terminal, rarely lateral, monoblastic, hyaline, subhyaline, pale brown, or brown, 3.5–13.5 × 2.5–4.5 µm. Conidia globose, subglobose, rarely broadly ellipsoid, hyaline, subhyaline, or brown, aseptate, rarely with 1–2 septa or muriformly septate, smooth or finely verrucose, thick-walled, 6.5–15 × 6–13.5 µm, sometimes germinating into hypha or produced intercalary, wall ca. 0.5–1.5 µm thick.

##### Culture characteristics.

Colonies on MEA erumpent, spreading, umbonate, grayish, reaching 1 mm diam. after 4 weeks at 6 °C, 4 mm diam. after 4 weeks at 15 °C, and 8 mm diam. after 4 weeks at 25 °C, with a velvety surface caused by abundant aerial mycelium, margin entire. Reverse black. Colonies on PDA erumpent, spreading, umbonate, grayish-brown, reaching 3 mm diam. after 4 weeks at 6 °C, 4 mm diam. after 4 weeks at 15 °C, and 7 mm diam. after 4 weeks at 25 °C, with a velvety surface caused by abundant aerial mycelium, margin finely undulate. Reverse black.

##### Notes.

**Figure 2.**
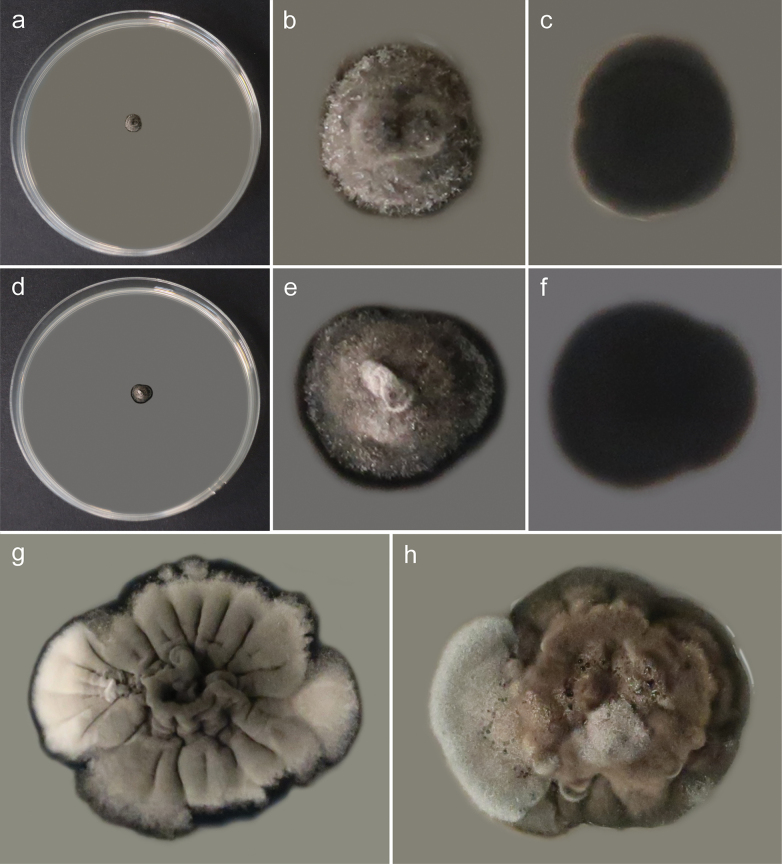
Morphology of cultures of *Szaferohyphaenigmatica* (strain G191 = CBS 152426) after 4 weeks of growth at 25 °C **a–c** general view, upper side and reverse side of colony on MEA **d–f** general view, upper side and reverse side of colony on PDA **g** general view of 15-week-old colony on MEA (to show difference in morphology) **h** general view of 15-week-old colony on PDA (to show difference in morphology).

Colonies photographed after 15 weeks of growth, depicted in Fig. 2g, h, are radially folded on MEA or folded on PDA, possess abundant aerial mycelium, grayish with white patches on MEA and brown with grayish patches on PDA, and are distinctly undulate at the margin.

Based on a megablast search of NCBI’s GenBank nucleotide database, the closest hits of the named species using the ITS sequence are *Pseudoarthrographisphlogis* (strain CPC 32759, GenBank MH327796; identities = 370/414 (89%), 11 gaps (2%)), *Oncopodiellatrigonella* (strain FMR 10788, GenBank KY853455; identities = 356/408 (87%), 11 gaps (2%)), and *Xylographaparallela* (voucher Z. Palice 22099 (PRM), GenBank MK778618; identities = 344/396 (87%), seven gaps (1%)). The closest hits of the named species using the LSU sequence are *Beaucarneamycesstellenboschensis* (strain CPC 45687, GenBank PP791445; identities = 826/873 (95%), four gaps (0%)), *Pseudoarthrographisphlogis* (strain CPC 32759, GenBank NG_064540; identities = 835/883 (95%), no gaps), and *Umbilicariahypococcinea* (strain A12, GenBank JQ739991; identities = 876/927 (94%), eight gaps (1%)). The closest hits using the SSU sequence are *Cophinformaatrovirens* (strain CSM_72, GenBank MF436134; identities = 857/870 (99%), no gaps), *Gloeopycnisprotuberans* (specimen DAOM 745762, GenBank NG_067652; identities = 857/870 (99%), no gaps), and *Botryosphaeriamamane* (strain CBS 117444, GenBank KF531821; identities = 857/870 (99%), no gaps). The closest hits using the *rpb2* sequence are *Shiraiabambusicola* (voucher SICAUCC 23-0005, GenBank OR424351; identities = 237/283 (84%), six gaps (2%)), *Lindraobtusa* (strain AFTOL-ID 5012, GenBank FJ238382; identities = 207/252 (82%), three gaps (1%)), and *Natonodosaspeciosa* (strain CLM-RV86, GenBank MH745150; identities = 217/266 (82%), six gaps (2%)). In the case of SSU and *rpb2*, the sequences of these two regions are not available for representatives of the most closely related genera, namely *Beaucarneamyces* and *Pseudoarthrographis*.

**Figure 3.**
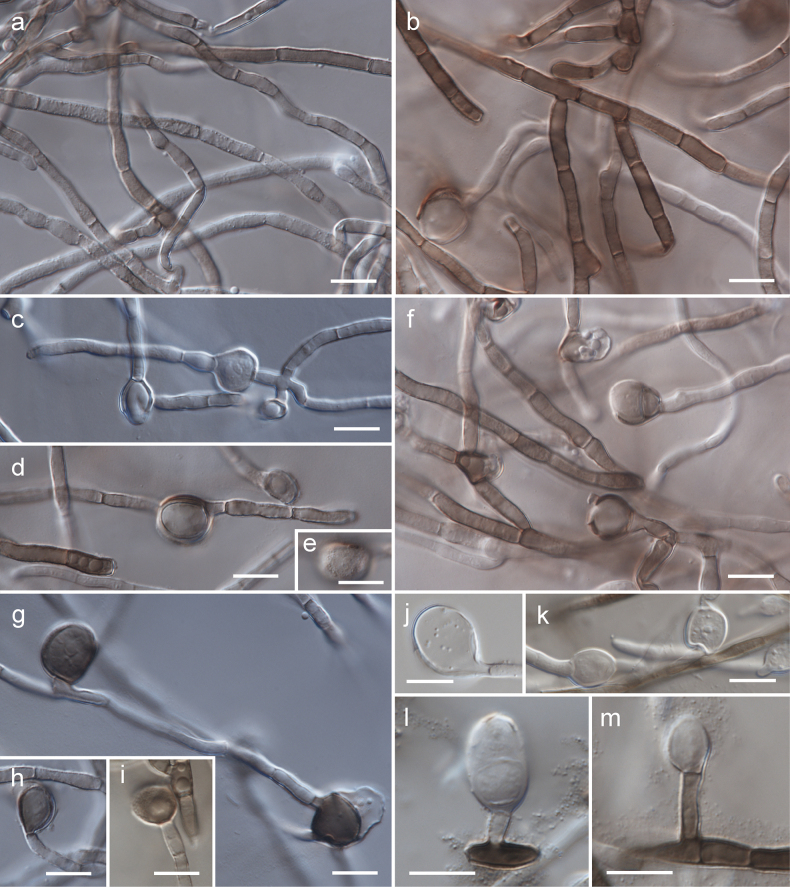
Morphology of *Szaferohyphaenigmatica* (strain G191 = CBS 152426, all on MEA) **a, b** subhyaline or brown, smooth or verrucose hyphae **c–e** subhyaline or brown intercalary conidia (figure **e** depicts the verrucose surface of the conidium) **f** hyphae and terminal conidia **g–m** hyaline or brown, lateral or terminal conidia emerging on conidiogenous cells (figure **i** depicts the verrucose surface of the conidium). Scale bars: 10 µm.

**Figure 4.**
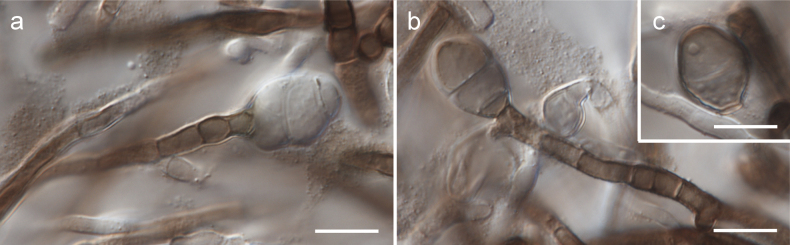
Morphology of *Szaferohyphaenigmatica* (strain G191 = CBS 152426, all on MEA) **a, b** terminal conidia emerging on conidiogenous cells **c** detached conidium. Scale bars: 10 µm.

## ﻿Discussion

In this study, morphological and phylogenetic analyses were conducted to identify the enigmatic fungal strain isolated from sooty mould biofilm on the surface of *Symphoricarposalbus* leaves. In consequence, the isolated fungus is described as a new genus and species, *Szaferohyphaenigmatica*, and assigned to the order Neodactylariales. Three genera were previously included in this order, namely *Beaucarneamyces*, *Neodactylaria*, and *Pseudoarthrographis* (Qiao et al. 2020; Crous et al. 2024), and *Szaferohypha* is morphologically different from all of them.

*Beaucarneamyces*, with type species *B.stellenboschensis*, was described from dead leaves of *Beaucarneastricta* in South Africa and is characterized by hyaline conidiophores reduced to conidiogenous cells that are polyblastic with several apical denticles. Conidia in this genus are solitary, fusoid-ellipsoid, hooked, 3-septate, hyaline but with pale brown central cells (Crous et al. 2024). *Neodactylaria* is typified with *N.obpyriformis* described from human bronchoalveolar lavage in the USA (Crous et al. 2017). The second species in this genus is *N.simaoensis*, described from submerged unidentified dicotyledonous leaves in China (Qiao et al. 2020). *Neodactylaria* is characterized by having straight or flexuous, brown conidiophores with polyblastic, sympodial, and denticle-like conidiogenous loci, which form solitary, obpyriform or rostrate, 0-1-septate, pale brown conidia (Crous et al. 2017). *Pseudoarthrographis*, with a type species *Pseudoarthrographisphlogis* described from *Phloxsubulata* in New Zealand, has smooth hyphae and smooth, 0–1-septate cylindrical arthroconidia with truncate ends produced in branched or unbranched chains. In culture it also forms smooth, globose chlamydospores occurring in chains (Crous et al. 2018).

Despite numerous strains obtained during our studies of sooty mould communities, only a single strain belonging to *Szaferohyphaenigmatica* was isolated. It is therefore unclear whether this species represents a very rare or accidental inhabitant of these communities and its main habitat is elsewhere.

The order Neodactylariales, with one family Neodactylariaceae, was originally described to accommodate only one genus, *Neodactylaria*, containing two species in China and the USA (Crous et al. 2017; Qiao et al. 2020). Recently, two monotypic genera, *Beaucarneamyces* and *Pseudoarthrographis*, containing species known in South Africa and New Zealand, respectively, were assigned to this order by Crous et al. (2024). Here, yet another monotypic genus, *Szaferohypha*, known from Poland, is added to Neodactylariales. Thus, currently this order includes fungi living in opposite corners of the world. The order Neodactylariales and family Neodactylariaceae were originally circumscribed based on characters known in the genus *Neodactylaria* (Qiao et al. 2020). They therefore need emendation by the features of the genera *Beaucarneamyces*, *Pseudoarthrographis*, and *Szaferohypha.* In our phylogenetic analysis, Neodactylariales is related to the order Oncopodiellales that is going to be described (Sun et al. 2024, preprint). Two genera, *Diplocladiella* and *Oncopodiella* (Hernández-Restrepo et al. 2017; Sun et al. 2024, preprint), assigned to two families, Diplocladiellaceae and Oncopodiellaceae, are included in this order (Sun et al. 2024, preprint). The relationships of Neodactylariales and Oncopodiellales are revealed here for the first time.

Sooty mould communities are an underexplored source of rare and undescribed species, of which many are probably extremophilic since they live in extreme environments (Chomnunti et al. 2014). They were previously studied using classical morphological methods (Hughes 1976) that are, however, inadequate to reveal their true diversity, and only recently started to study in modern ways using morphological and molecular methods, though mainly in tropics (Chomnunti et al. 2012, 2014) and much more rarely in temperate regions (Flessa et al. 2012, 2021). In the course of our ongoing studies of sooty mould communities covering the surface of leaves/needles of ornamental plants in Poland, i.e., in the temperate climate, about 190 species were isolated from these communities (M. Piątek et al. unpublished data), being either as constant or accidental colonizers, of which many are undescribed. These include four new *Rachicladosporium* species (Piątek et al. 2023), a new *Lapidomyces* species (Crous et al. 2023b), a new *Xenoramularia* species (Crous et al. 2023a), a new *Pseudopezicula* species (Crous et al. 2024), and many more that are waiting for description.

## Supplementary Material

XML Treatment for Neodactylariales

XML Treatment for Neodactylariaceae

XML Treatment for Szaferohypha

XML Treatment for Szaferohypha enigmatica

## ﻿Additional information

### Author ORCIDs

Marcin Piątek https://orcid.org/0000-0002-4968-2861

Monika Stryjak-Bogacka https://orcid.org/0000-0003-2845-9975

Paweł Czachura https://orcid.org/0000-0002-3562-8776

### Data availability

The data that support the findings of this study are available in GenBank (https://www.ncbi.nlm.nih.gov/genbank/) and in culture collections and fungal herbaria, as shown in Suppl. material 1 and the text.

## Supplementary materials

Supplementary material 1 List of species with strain/specimen and GenBank accession numbers used in phylogenetic analysis Data type docx https://binary.pensoft.net/file/1208440This dataset is made available under the Open Database License (http://opendatacommons.org/licenses/odbl/1.0/). The Open Database License (ODbL) is a license agreement intended to allow users to freely share, modify, and use this Dataset while maintaining this same freedom for others, provided that the original source and author(s) are credited.Marcin Piątek, Monika Stryjak-Bogacka, Paweł Czachura

## References

[B1] Abdollahzadeh J Groenewald JZ Coetzee MPA Wingfield MJ Crous PW (2020) Evolution of lifestyles in Capnodiales. Studies in Mycology 95: 381–414. 10.1016/j.simyco.2020.02.004 32855743 PMC7426231

[B2] Barreto GG Souza-Motta CM Silva GA Groenewald JZ Crous PW Bezerra JDP (2024) Meristematic and meristematic-like fungi in Dothideomycetes. Fungal Systematics and Evolution 14: 77–88. 10.3114/fuse.2024.14.05 PMC11736082 39830295

[B3] Chomnunti P Bhat DJ Jones EBG Chukeatirote E Bahkali AH Hyde KD (2012) Trichomeriaceae, a new sooty mould family of Chaetothyriales. Fungal Diversity 56(1): 63–76. 10.1007/s13225-012-0197-2

[B4] Chomnunti P Hongsanan S Aguirre-Hudson B Tian Q Peršoh D Dhami MK Alias AS Xu J Liu XH Stadler M Hyde KD (2014) The sooty moulds. Fungal Diversity 66(1): 1–36. 10.1007/s13225-014-0278-5

[B5] Coleine C Pombubpa N Zucconi L Onofri S Stajich JE Selbmann L (2020) Endolithic fungal species markers for harshest conditions in the McMurdo Dry Valleys, Antarctica. Life (Basel, Switzerland) 10(2): 13. 10.3390/life10020013 32041249 PMC7175349

[B6] Crous PW Wingfield MJ Burgess TI Hardy GESJ Barber PA Alvarado P Barnes CW Buchanan PK Heykoop M Moreno G Thangavel R van der Spuy S Barili A Barrett S Cacciola SO Cano-Lira JF Crane C Decock C Gibertoni TB Guarro J Guevara-Suarez M Hubka V Kolařík M Lira CRS Ordoñez ME Padamsee M Ryvarden L Soares AM Stchigel AM Sutton DA Vizzini A Weir BS Acharya K Aloi F Baseia IG Blanchette RA Bordallo JJ Bratek Z Butler T Cano-Canals J Carlavilla JR Chander J Cheewangkoon R Cruz RHSF da Silva M Dutta AK Ercole E Escobio V Esteve-Raventós F Flores JA Gené J Góis JS Haines L Held BW Jung MH Hosaka K Jung T Jurjević Ž Kautman V Kautmanova I Kiyashko AA Kozanek M Kubátová A Lafourcade M La Spada F Latha KPD Madrid H Malysheva EF Manimohan P Manjón JL Martín MP Mata M Merényi Z Morte A Nagy I Normand AC Paloi S Pattison N Pawłowska J Pereira OL Petterson ME Picillo B Raj KNA Roberts A Rodríguez A Rodríguez-Campo FJ Romański M Ruszkiewicz-Michalska M Scanu B Schena L Semelbauer M Sharma R Shouche YS Silva V Staniaszek-Kik M Stielow JB Tapia C Taylor PWJ Toome-Heller M Vabeikhokhei JMC van Diepeningen AD Van Hoa N (2017) Fungal Planet description sheets: 558–624. Persoonia 38(1): 240–384. 10.3767/003158517X698941 29151634 PMC5645186

[B7] Crous PW Wingfield MJ Burgess TI Hardy GESJ Gené J Guarro J Baseia IG García D Gusmão LFP Souza-Motta CM Thangavel R Adamčík S Barili A Barnes CW Bezerra JDP Bordallo JJ Cano-Lira JF de Oliveira RJV Ercole E Hubka V Iturrieta-González I Kubátová A Martín MP Moreau PA Morte A Ordoñez ME Rodríguez A Stchigel AM Vizzini A Abdollahzadeh J Abreu VP Adamčíková K Albuquerque GMR Alexandrova AV Álvarez Duarte E Armstrong-Cho C Banniza S Barbosa RN Bellanger JM Bezerra JL Cabral TS Caboň M Caicedo E Cantillo T Carnegie AJ Carmo LT Castañeda-Ruiz RF Clement CR Čmoková A Conceição LB Cruz RHSF Damm U da Silva BDB da Silva GA da Silva RMF Santiago ALCMA de Oliveira LF de Souza CAF Déniel F Dima B Dong G Edwards J Félix CR Fournier J Gibertoni TB Hosaka K Iturriaga T Jadan M Jany J-L Jurjević Ž Kolařík M Kušan I Landell MF Leite Cordeiro TR Lima DX Loizides M Luo S Machado AR Madrid H Magalhães OMC Marinho P Matočec N Mešić A Miller AN Morozova OV Neves RP Nonaka K Nováková A Oberlies NH Oliveira-Filho JRC Oliveira TGL Papp V Pereira OL Perrone G Peterson SW Pham THG Raja HA (2018) Fungal Planet description sheets: 716–784. Persoonia 40(1): 240–393. 10.3767/persoonia.2018.40.10 30505003 PMC6146637

[B8] Crous PW Costa MM Kandemir H Vermaas M Vu D Zhao L Abell SE Arumugam E Flakus A Jurjević Ž Kaliyaperumal M Mahadevakumar S Marney TS Murugadoss R Shivas RG Tan YP Wingfield MJ Danteswari C Darmostuk V Denchev TT Etayo J Gené J Gunaseelan S Hubka V Illescas T Jansen GM Kezo K Kumar S Larsson E Mufeeda KT Piątek M Rodriguez-Flakus P Sarma PVSRN Stryjak-Bogacka M Torres-Garcia D Vauras J Acal DA Akulov A Alhudaib K Asif M Balashov S Baral HO Baturo-Cieśniewska A Begerow D Beja-Pereira A Bianchinotti MV Bilański P Chandranayaka S Chellappan N Cowan DA Custódio FA Czachura P Delgado G De Silva NI Dijksterhuis J Dueñas M Eisvand P Fachada V Fournier J Fritsche Y Fuljer F Ganga KGG Guerra MP Hansen K Hywel-Jones N Ismail AM Jacobs CR Jankowiak R Karich A Kemler M Kisło K Klofac W Krisai-Greilhuber I Latha KPD Lebeuf R Lopes ME Lumyong S Maciá-Vicente JG Maggs-Kölling G Magistà D Manimohan P Martín MP Mazur E Mehrabi-Koushki M Miller AN Mombert A Ossowska EA Patejuk K Pereira OL Piskorski S Plaza M Podile AR Polhorský A Pusz W Raza M Ruszkiewicz-Michalska M Saba M Sánchez RM Singh R Śliwa L Smith ME Stefenon VM Strašiftáková D Suwannarach N Szczepańska K Telleria MT Tennakoon DS Thines M Thorn RG Urbaniak J van der Vegte M Vasan V Vila-Viçosa C Voglmayr H Wrzosek M Zappelini J Groenewald JZ (2023a) Fungal Planet description sheets: 1550–1613. Persoonia 51(1): 280–417. 10.3767/persoonia.2023.51.08 38665977 PMC11041897

[B9] Crous PW Osieck ER Shivas RG Tan YP Bishop-Hurley SL Esteve-Raventós F Larsson E Luangsa-ard JJ Pancorbo F Balashov S Baseia IG Boekhout T Chandranayaka S Cowan DA Cruz RHSF Czachura P De la Peña-Lastra S Dovana F Drury B Fell J Flakus A Fotedar R Jurjević Ž Kolecka A Mack J Maggs-Kölling G Mahadevakumar S Mateos A Mongkolsamrit S Noisripoom W Plaza M Overy DP Piątek M Sandoval-Denis M Vauras J Wingfield MJ Abell SE Ahmadpour A Akulov A Alavi F Alavi Z Altés A Alvarado P Anand G Ashtekar N Assyov B Banc-Prandi G Barbosa KD Barreto GG Bellanger JM Bezerra JL Bhat DJ Bilański P Bose T Bozok F Chaves J Costa-Rezende DH Danteswari C Darmostuk V Delgado G Denman S Eichmeier A Etayo J Eyssartier G Faulwetter S Ganga KGG Ghosta Y Goh J Góis JS Gramaje D Granit L Groenewald M Gulden G Gusmão LFP Hammerbacher A Heidarian Z Hywel-Jones N Jankowiak R Kaliyaperumal M Kaygusuz O Kezo K Khonsanit A Kumar S Kuo CH Læssøe T Latha KPD Loizides M Luo SM Maciá-Vicente JG Manimohan P Marbach PAS Marinho P Marney TS Marques G Martín MP Miller AN Mondello F Moreno G Mufeeda KT Mun HY Nau T Nkomo T Okrasińska A Oliveira JPAF Oliveira RL Ortiz DA Pawłowska J Pérez-De-Gregorio MÀ Podile AR Portugal A Privitera N Rajeshkumar KC Rauf I Rian B Rigueiro-Rodríguez A Rivas-Torres GF Rodriguez-Flakus P Romero-Gordillo M Saar I Saba M Santos CD Sarma PVSRN Siquier JL Sleiman S Spetik M Sridhar KR Stryjak-Bogacka M Szczepańska K Taşkın H Tennakoon DS Thanakitpipattana D Trovão J Türkekul İ van Iperen AL van ’t Hof P Vasquez G Visagie CM Wingfield BD Wong PTW Yang WX Yarar M Yarden O Yilmaz N Zhang N Zhu YN Groenewald JZ (2023b) Fungal Planet description sheets: 1478–1549. Persoonia 50(1): 158–310. 10.3767/persoonia.2023.50.05 38567263 PMC10983837

[B10] Crous PW Jurjević Z Balashov S De la Peña-Lastra S Mateos A Pinruan U Rigueiro-Rodríguez A Osieck ER Altés A Czachura P Esteve-Raventós F Gunaseelan S Kaliyaperumal M Larsson E Luangsa-ard JJ Moreno G Pancorbo F Piątek M Sommai S Somrithipol S Asif M Delgado G Flakus A Illescas T Kezo K Khamsuntorn P Kubátová A Labuda R Lavoise C Lebel T Lueangjaroenkit P Maciá-Vicente JG Paz A Saba M Shivas RG Tan YP Wingfield MJ Aas T Abramczyk B Ainsworth AM Akulov A Alvarado P Armada F Assyov B Avchar R Avesani M Bezerra JL Bhat JD Bilański P Bily DS Boccardo F Bozok F Campos JC Chaimongkol S Chellappan N Costa MM Dalecká M Darmostuk V Daskalopoulos V Dearnaley J Dentinger BTM De Silva NI Dhotre D Carlavilla JR Doungsa-ard C Dovana F Erhard A Ferro LO Gallegos SC Giles CE Gore G Gorfer M Guard FE Hanson SA Haridev P Jankowiak R Jeffers SN Kandemir H Karich A Kisło K Kiss L Krisai-Greilhuber I Latha KPD Lorenzini M Lumyong S Manimohan P Manjón JL Maula F Mazur E Mesquita NLS Młynek K Mongkolsamrit S Morán P Murugadoss R Nagarajan M Nalumpang S Noisripoom W Nosalj S Novaes QS Nowak M Pawłowska J Peiger M Pereira OL Pinto A Plaza M Polemis E Polhorský A Ramos DO Raza M Rivas-Ferreiro M Rodriguez-Flakus P Ruszkiewicz-Michalska M Sánchez A Santos A Schüller A Scott PA Şen İ Shelke D Śliwa L Solheim H Sonawane H Strašiftáková D Stryjak-Bogacka M Sudsanguan M Suwannarach N Suz LM Syme K Taşkın H Tennakoon DS Tomka P Vaghefi N Vasan V Vauras J Wiktorowicz D Villarreal M Vizzini A Wrzosek M Yang X Yingkunchao W Zapparoli G Zervakis GI Groenewald JZ (2024) Fungal Planet description sheets: 1614–1696. Fungal Systematics and Evolution 13: 183–439. 10.3114/fuse.2024.13.11 39140100 PMC11320056

[B11] Czachura P Janik P (2024) [2023] *Lophiumarboricola* (Mytilinidiales, Ascomycota) from conifer resins. Plant and Fungal Systematics 69(1): 1–6. 10.35535/pfsyst-2024-0001

[B12] Czachura P Owczarek-Kościelniak M Piątek M (2021) *Salinomycespolonicus*: A moderately halophilic kin of the most extremely halotolerant fungus *Hortaeawerneckii*. Fungal Biology 125(6): 459–468. 10.1016/j.funbio.2021.01.003 34024593

[B13] Darriba D Posada D Kozlov AM Stamatakis A Morel B Flouri T (2020) ModelTest-NG: A new and scalable tool for the selection of DNA and protein evolutionary models. Molecular Biology and Evolution 37(1): 291–294. 10.1093/molbev/msz189 31432070 PMC6984357

[B14] Flessa F Peršoh D Rambold G (2012) Annuality of Central European deciduous tree leaves delimits community development of epifoliar pigmented fungi. Fungal Ecology 5(5): 554–561. 10.1016/j.funeco.2011.12.005

[B15] Flessa F Harjes J Cáceres MES Rambold G (2021) Comparative analyses of sooty mould communities from Brazil and Central Europe. Mycological Progress 20(7): 869–887. 10.1007/s11557-021-01700-0

[B16] Hernández-Restrepo M Gené J Castañeda-Ruiz RF Mena-Portales J Crous PW Guarro J (2017) Phylogeny of saprobic microfungi from Southern Europe. Studies in Mycology 86: 53–97. 10.1016/j.simyco.2017.05.002 28626275 PMC5470572

[B17] Hongsanan S Sánchez-Ramírez S Crous PW Ariyawansa HA Zhao RL Hyde KD (2016) The evolution of fungal epiphytes. Mycosphere 7(11): 1690–1712. 10.5943/mycosphere/7/11/6

[B18] Hongsanan S Hyde KD Phookamsak R Wanasinghe DN McKenzie EHC Sarma VV Boonmee S Lücking R Bhat DJ Liu NG Tennakoon DS Pem D Karunarathna A Jiang SH Jones EBG Phillips AJL Manawasinghe IS Tibpromma S Jayasiri SC Sandamali DS Jayawardena RS Wijayawardene NN Ekanayaka AH Jeewon R Lu YZ Dissanayake AJ Zeng XY Luo ZL Tian Q Phukhamsakda C Thambugala KM Dai DQ Chethana KWT Samarakoon MC Ertz D Bao DF Doilom M Liu JK Pérez-Ortega S Suija A Senwanna C Wijesinghe SN Konta S Niranjan M Zhang SN Ariyawansa HA Jiang HB Zhang JF Norphanphoun C de Silva NI Thiyagaraja V Zhang H Bezerra JDP Miranda-González R Aptroot A Kashiwadani H Harishchandra D Sérusiaux E Aluthmuhandiram JVS Abeywickrama PD Devadatha B Wu HX Moon KH Gueidan C Schumm F Bundhun D Mapook A Monkai J Chomnunti P Suetrong S Chaiwan N Dayarathne MC Yang J Rathnayaka AR Bhunjun CS Xu JC Zheng JS Liu G Feng Y Xie N (2020a) Refined families of Dothideomycetes: Dothideomycetidae and Pleosporomycetidae. Mycosphere 11(1): 1553–2107. 10.5943/mycosphere/11/1/13

[B19] Hongsanan S Hyde KD Phookamsak R Wanasinghe DN McKenzie EHC Sarma VV Lücking R Boonmee S Bhat JD Liu NG Tennakoon DS Pem D Karunarathna A Jiang SH Jones GEB Phillips AJL Manawasinghe IS Tibpromma S Jayasiri SC Sandamali D Jayawardena RS Wijayawardene NN Ekanayaka AH Jeewon R Lu YZ Phukhamsakda C Dissanayake AJ Zeng XY Luo ZL Tian Q Thambugala KM Dai D Samarakoon MC Chethana KWT Ertz D Doilom M Liu JK Pérez-Ortega S Suija A Senwanna C Wijesinghe SN Niranjan M Zhang SN Ariyawansa HA Jiang HB Zhang JF Norphanphoun C de Silva NI Thiyagaraja V Zhang H Bezerra JDP Miranda-González R Aptroot A Kashiwadani H Harishchandra D Sérusiaux E Abeywickrama PD Bao DF Devadatha B Wu HX Moon KH Gueidan C Schumm F Bundhun D Mapook A Monkai J Bhunjun CS Chomnunti P Suetrong S Chaiwan N Dayarathne MC Yang J Rathnayaka AR Xu JC Zheng J Liu G Feng Y Xie N (2020b) Refined families of Dothideomycetes: Orders and families incertae sedis in Dothideomycetes. Fungal Diversity 105(1): 17–318. 10.1007/s13225-020-00462-6

[B20] Hughes SJ (1976) Sooty moulds. Mycologia 68(4): 693–820. 10.1080/00275514.1976.12019958

[B21] Katoh K Kuma K Toh H Miyata T (2005) MAFFT version 5: Improvement in accuracy of multiple sequence alignment. Nucleic Acids Research 33(2): 511–518. 10.1093/nar/gki198 15661851 PMC548345

[B22] Kolařík M Stępniewska H Jankowiak R (2021) Taxonomic revision of the acidophilic genus *Acidiella* (Dothideomycetes, Capnodiales) with a description of new species from Poland. Plant Systematics and Evolution 307(3): 38. 10.1007/s00606-021-01753-4

[B23] Kozlov AM Darriba D Flouri T Morel B Stamatakis A (2019) RAxML-NG: A fast, scalable, and user-friendly tool for maximum likelihood phylogenetic inference. Bioinformatics (Oxford, England) 35(21): 4453–4455. 10.1093/bioinformatics/btz305 31070718 PMC6821337

[B24] Liu YL Whelen S Hall BD (1999) Phylogenetic relationships among ascomycetes: Evidence from an RNA polymerase II subunit. Molecular Biology and Evolution 16(12): 1799–1808. 10.1093/oxfordjournals.molbev.a026092 10605121

[B25] Maharachchikumbura SSN Wanasinghe DN Cheewangkoon R Al-Sadi AM (2021) Uncovering the hidden taxonomic diversity of fungi in Oman. Fungal Diversity 106(1): 229–268. 10.1007/s13225-020-00467-1

[B26] Nagahama T Takahashi E Nagano Y Abdel-Wahab MA Miyazaki M (2011) Molecular evidence that deep-branching fungi are major fungal components in deep-sea methane cold-seep sediments. Environmental Microbiology 13(8): 2359–2370. 10.1111/j.1462-2920.2011.02507.x 21605311

[B27] Pem D Hyde KD McKenzie EHC Hongsanan S Wanasinghe DN Boonmee S Darmostuk V Bhat JD Tian Q Htet ZH Senanayake IC Niranjan M Sarma VV Doilom M Dong W (2024) A comprehensive overview of genera in Dothideomycetes. Mycosphere 15(1): 2175–4568. 10.5943/mycosphere/15/1/18

[B28] Phillips AJL Alves A Abdollahzadeh J Slippers B Wingfield MJ Groenewald JZ Crous PW (2013) The Botryosphaeriaceae: Genera and species known from culture. Studies in Mycology 76: 51–167. 10.3114/sim0021 24302790 PMC3825232

[B29] Phillips AJL Hyde KD Alves A Liu JK (2019) Families in Botryosphaeriales: A phylogenetic, morphological and evolutionary perspective. Fungal Diversity 94(1): 1–22. 10.1007/s13225-018-0416-6

[B30] Piątek M Stryjak-Bogacka M Czachura P Owczarek-Kościelniak M (2023) The genus *Rachicladosporium*: Introducing new species from sooty mould communities and excluding cold adapted species. Scientific Reports 13(1): 22795. 10.1038/s41598-023-49696-9 38129458 PMC10739867

[B31] Piątek M Stryjak-Bogacka M Czachura P (2024) Arthrocatenales, a new order of extremophilic fungi in the Dothideomycetes. MycoKeys 108: 47–74. 10.3897/mycokeys.108.128033 39220356 PMC11362667

[B32] Qiao M Zheng H Lv R Yu Z (2020) Neodactylariales, Neodactylariaceae (Dothideomycetes, Ascomycota): New order and family, with a new species from China. MycoKeys 73: 69–85. 10.3897/mycokeys.73.54054 32994703 PMC7501314

[B33] Quaedvlieg W Binder M Groenewald JZ Summerell BA Carnegie AJ Burgess TI Crous PW (2014) Introducing the Consolidated Species Concept to resolve species in the Teratosphaeriaceae. Persoonia 33(1): 1–40. 10.3767/003158514X681981 25737591 PMC4312929

[B34] Rojas-Jimenez K Grossart HP Cordes E Cortés J (2020) Fungal communities in sediments along a depth gradient in the Eastern Tropical Pacific. Frontiers in Microbiology 11: 575207. 10.3389/fmicb.2020.575207 PMC7681244 33240232

[B35] Shen M Zhang JQ Zhao LL Groenewald JZ Crous PW Zhang Y (2020) Venturiales. Studies in Mycology 96: 185–308. 10.1016/j.simyco.2020.03.001 32904190 PMC7452091

[B36] Sun YR Hyde KD Liu NG Jayawardena RS Wijayawardene NN Ma J Zhang Q Al-Otibi F Wang Y (2024) Micro-fungi on medicinal plants in southern China and northern Thailand, 03 October 2024, PREPRINT (Version 1) [available at Research Square] 10.21203/rs.3.rs-4813665/v1

[B37] Thambugala KM Ariyawansa HA Li YM Boonmee S Hongsanan S Tian Q Singtripop C Bhat DJ Camporesi E Jayawardena R Liu ZY Xu JC Chukeatirote E Hyde KD (2014) Dothideales. Fungal Diversity 68(1): 105–158. 10.1007/s13225-014-0303-8

[B38] Videira SIR Groenewald JZ Nakashima C Braun U Barreto RW de Wit PJGM Crous PW (2017) Mycosphaerellaceae – chaos or clarity? Studies in Mycology 87(1): 257–421. 10.1016/j.simyco.2017.09.003 PMC5693839 29180830

[B39] Vilgalys R Hester M (1990) Rapid genetic identification and mapping of enzymatically amplified ribosomal DNA from several *Cryptococcus* species. Journal of Bacteriology 172(8): 4238–4246. 10.1128/jb.172.8.4238-4246.1990 2376561 PMC213247

[B40] White TJ Bruns T Lee S Taylor J (1990) Amplification and direct sequencing of fungal ribosomal RNA genes for phylogenetics. In: Innis MA Gelfand DH Sninsky JJ White TJ (Eds) PCR Protocols: a guide to methods and applications.Academic Press, San Diego, 315–322. 10.1016/B978-0-12-372180-8.50042-1

[B41] Wijayawardene NN Crous PW Kirk PM Hawksworth DL Boonmee S Braun U Dai DQ D’souza MJ Diederich P Dissanayake A Doilom M Hongsanan S Jones EBG Groenewald JZ Jayawardena R Lawrey JD Liu JK Lücking R Madrid H Manamgoda DS Muggia L Nelsen MP Phookamsak R Suetrong S Tanaka K Thambugala KM Wanasinghe DN Wikee S Zhang Y Aptroot A Ariyawansa HA Bahkali AH Bhat JD Gueidan C Chomnunti P de Hoog GS Knudsen K Li WJ McKenzie EHC Miller AN Mortimer PE Phillips AJL Piątek M Raja HA Shivas RG Slippers B Taylor JE Tian Q Wang Y Woudenberg JHC Cai L Jaklitsch WM Hyde KD (2014) Naming and outline of Dothideomycetes–2014 including proposals for the protection or suppression of generic names. Fungal Diversity 69(1): 1–55. 10.1007/s13225-014-0309-2 27284275 PMC4896388

[B42] Wijayawardene NN Hyde KD Al-Ani LKT Tedersoo L Haelewaters D Rajeshkumar KC Zhao RL Aptroot A Leontyev DV Saxena RK Tokarev YS Dai DQ Letcher PM Stephenson SL Ertz D Lumbsch HT Kukwa M Issi IV Madrid H Phillips AJL Selbmann L Pfliegler WP Horváth E Bensch K Kirk PM Kolaříková K Raja HA Radek R Papp V Dima B Ma J Malosso E Takamatsu S Rambold G Gannibal PB Triebel D Gautam AK Avasthi S Suetrong S Timdal E Fryar SC Delgado G Réblová M Doilom M Dolatabadi S Pawłowska J Humber RA Kodsueb R Sánchez-Castro I Goto BT Silva DKA de Souza FA Oehl F da Silva GA Silva IR Błaszkowski J Jobim K Maia LC Barbosa FR Fiuza PO Divakar PK Shenoy BD Castañeda-Ruiz RF Somrithipol S Lateef AA Karunarathna SC Tibpromma S Mortimer PE Wanasinghe DN Phookamsak R Xu J Wang Y Tian F Alvarado P Li DW Kušan I Matočec N Maharachchikumbura SSN Papizadeh M Heredia G Wartchow F Bakhshi M Boehm E Youssef N Hustad VP Lawrey JD Santiago ALCMA Bezerra JDP Souza-Motta CM Firmino AL Tian Q Houbraken J Hongsanan S Tanaka K Dissanayake AJ Monteiro JS Grossart HP Suija A Weerakoon G Etayo J Tsurykau A Vázquez V Mungai P Damm U Li QR Zhang H Boonmee S Lu YZ Becerra AG Kendrick B Brearley FQ Motiejūnaitė J Sharma B Khare R Gaikwad S Wijesundara DSA Tang LZ He MQ Flakus A Rodriguez-Flakus P Zhurbenko MP McKenzie EHC Stadler M Bhat DJ Liu JK Raza M Jeewon R Nassonova ES Prieto M Jayalal RGU Erdoğdu M Yurkov A Schnittler M Shchepin ON Novozhilov YK Silva-Filho AGS Liu P Cavender JC Kang Y Mohammad S Zhang LF Xu RF Li YM Dayarathne MC Ekanayaka AH Wen TC Deng CY Pereira OL Navathe S Hawksworth DL Fan XL Dissanayake LS Kuhnert E Grossart HP Thines M (2020) Outline of *Fungi* and fungus-like taxa. Mycosphere 11(1): 1060–1456. 10.5943/mycosphere/11/1/8

[B43] Yang T Groenewald JZ Cheewangkoon R Jami F Abdollahzadeh J Lombard L Crous PW (2017) Families, genera, and species of Botryosphaeriales. Fungal Biology 121(4): 322–346. 10.1016/j.funbio.2016.11.001 28317538

[B44] Zhang Z Schwartz S Wagner L Miller W (2000) A greedy algorithm for aligning DNA sequences. Journal of Computational Biology 7(1–2): 203–214. 10.1089/10665270050081478 10890397

[B45] Zhang Y Crous PW Schoch CL Hyde KD (2012) Pleosporales. Fungal Diversity 53(1): 1–221. 10.1007/s13225-011-0117-x 23097638 PMC3477819

