# Supplementary material for: ﻿Emended Neodactylariales (Dothideomycetes): Szaferohypha gen. nov. and phylogenetically related genera
Source: MycoKeys. 2024 Dec 27;111:211–28. doi: 10.3897/mycokeys.111.139620 (PMC11699514; doi:10.3897/mycokeys.111.139620)
Supplement: Supplementary material 1 — List of species with strain/specimen and GenBank accession numbers used in phylogenetic analysis [file mycokeys-111-211-s001.docx]

**Supplementary Table 1.** List of species with strain/specimen and GenBank accession numbers used in phylogenetic analyses.

| **Species** | **Strain/specimen** | **GenBank acc. no.** | | | |
| --- | --- | --- | --- | --- | --- |
|  |  | **ITS** | **LSU** | **SSU** | ***rpb2*** |
| *Abrothallus acetabuli* | SPO308 | KF816165 | KF816232 | KF816215 | KF816194 |
| *Abrothallus buellianus* | SPO303 | KF816166 | KF816234 | KF816217 | KF816195 |
| *Abrothallus hypotrachynae* | SPO302 | KF816167 | KF816233 | KF816218 | KF816196 |
| *Acrospermum adeanum* | M133 | — | EU940104 | EU940031 | — |
| *Acrospermum compressum* | M151 | — | EU940084 | EU940012 | — |
| *Acrospermum gramineum* | M152 | — | EU940085 | EU940013 | — |
| *Acrospermum longisporium* | MFLU 17-2849 | — | NG_064506 | NG_065734 | — |
| *Aliquandostipite khaoyaiensis* | CBS 118232 | — | GU301796 | NG_016494 | — |
| *Alloarthopyrenia italica* | MFLU 15-0399 | NR_153558 | KX655550 | — | — |
| *Aplosporella africana* | CBS 121777 | NR_136995 | EU101380 | NG_065027 | — |
| *Aplosporella papillata* | CBS 121780 | NR_136997 | EU101383 | KF766284 | — |
| *Aplosporella prunicola* | CBS 121167 | NR_119642 | KF766315 | NG_063004 | — |
| *Arthrographis kalrae* | CBS 693.77 | NR_126108 | NG_056932 | — | — |
| *Arthrographis longispora* | CBS 135935 | — | MH877597 | — | — |
| *Asterina cynometrae* | MFLU 13-0373 | — | NG_057120 | — | — |
| *Asterina phenacis* | TH 589 | — | GU586217 | — | — |
| *Asterina weinmanniae* | TH 592 | — | GU586218 | — | — |
| *Asterodiscus tamaricis* | CBS 136919 | NR_153544 | KU234101 | NG_061222 | KU234116 |
| *Asterotexis cucurbitacearum* | VIC 42814 | — | NG_057054 | — | — |
| *Aureobasidium lini* | CBS 125.21 | NR_147320 | NG_056962 | EU707925 | KT693984 |
| *Banhegyia* cf. *setispora* | G.M. 2015-04-29 | KY654708 | KY654708 | — | — |
| *Banhegyia setispora* | G.M. 2014-05-25.1 | KY654747 | KY654747 | — | — |
| *Beaucarneamyces stellenboschensis* | CPC 45687 | PP791416 | PP791445 | — | — |
| *Botryobambusa fusicoccum* | MFLUCC 11-0143 | NR_111793 | NG_042724 | — | — |
| *Botryosphaeria dothidea* | CBS 115476 | KF766151 | NG_027577 | NG_062738 | DQ677944 |
| *Botryosphaeria pseudoramosa* | CGMCC 3.18739 | KX277989 | MF410031 | MF410229 | MF410140 |
| *Botryosphaeria qingyuanensis* | CGMCC 3.18742 | KX278000 | MF410042 | MF410240 | MF410151 |
| *Botryosphaeria wangensis* | CGMCC 3.18744 | KX278002 | MF410044 | MF410242 | MF410153 |
| *Brachiosphaera tropicalis* | SS2523 | FJ887923 | JN819284 | JN819287 | — |
| *Capnodium coffeae* | CBS 147.52 | MH856967 | DQ247800 | DQ247808 | KT216519 |
| *Catinella olivacea* | UAMH 10679 | DQ915483 | EF622212 | DQ915484 | — |
| *Cercospora beticola* | CBS 116456 | NR_121315 | DQ678091 | NG_062715 | KT216555 |
| *Cladoriella eucalypti* | CPC 10953 | DQ195778 | DQ195790 | DQ195801 | — |
| *Cladoriella kinglakensis* | CPC 32730 | NR_156396 | NG_059055 | — | — |
| *Clypeococcum psoromatis* | Ertz 19259 | — | KT383790 | — | — |
| *Collemopsidium angermannicum* | s1473 | — | KU556871 | KU556972 | KU556864 |
| *Collemopsidium* cf. *foveolatum* | RO27 | — | KU556973 | KU556967 | — |
| *Collemopsidium* cf. *halodytes* | RO26 | — | KU556869 | KU556966 | KU556862 |
| *Coniosporium* *apollinis* | CBS 109860 | — | GU250899 | GU250920 | — |
| *Cryomyces* *minteri* | CCFEE 5187 | — | GU250369 | KC315858 | — |
| *Cryomyces* *montanus* | CCFEE 5476 | KC315849 | GU250394 | GU250352 | — |
| *Diplocladiella scalaroides* | 18C-46 | OP377842 | OP377930 | OP378017 | — |
| *Diplocladiella hannanensis* | YRS-2024a | PQ212579 | PQ208461 | — | — |
| *Dissoconium aciculare* | CBS 204.89 | AY725520 | GU214419 | GU214523 | KX288435 |
| *Dothidea insculpta* | CBS 189.58 | AF027764 | DQ247802 | DQ247810 | DQ247792 |
| *Dothidea oleae* | CBS 615.72 | KU728511 | EU754148 | EU754049 | — |
| *Dothiora cannabinae* | CBS 737.71 | NR_144904 | DQ470984 | — | DQ470936 |
| *Dyfrolomyces maolanensis* | GZCC 16-0102 | — | KY111905 | KY111906 | — |
| *Dyfrolomyces sinensis* | MFLUCC 17-1344 | — | MG836699 | MG836700 | — |
| *Dyfrolomyces thamplaensis* | MFLUCC 15-0635 | — | KX925435 | KX925436 | — |
| *Elsinoe centrolobii* | CBS 222.50 | NR_148132 | KX886969 | NG_062717 | KX887089 |
| *Elsinoe phaseoli* | CBS 165.31 | NR_148161 | DQ678095 | NG_062718 | KX887144 |
| *Encephalographa elisae* | EB 0347 | — | GU397343 | GU397358 | — |
| *Endomelanconiopsis endophytica* | CBS 120397 | NR_156272 | NG_059196 | KF766249 | KX463981 |
| *Eremithallus costaricensis* | F:Lücking 15683 | — | EU622918 | — | MN562644 |
| *Flavobathelium epiphyllum* | MPN67 | — | GU327717 | JN887382 | — |
| *Fusicladium africanum* | CPC 12828 | EU035423 | EU035423 | — | — |
| *Gloniopsis* *praclonga* | CBS 112415 | — | — | FJ161134 | — |
| *Glyphium elatum* | EB 0365 | KM220945 | KM220939 | KM220936 | — |
| *Glyphium elatum* | EB 0342 | KM220944 | KM220938 | KM220935 | — |
| *Holmiella junipericola* | MFLUCC 18-0503 | MH188902 | MH188900 | MH188901 | — |
| *Holmiella junipericola* | SQUCC 15186 | MW077142 | MW077151 | MW077160 | MW276074 |
| *Holmiella juniperi-semiglobosae* | MFLUCC 17-1955 | MH188905 | MH188903 | MH188904 | — |
| *Holmiella sabina* | G.M. 2015-04-29.2 | KY486750 | KY486750 | — | — |
| *Holmiella sabina* | G.M. 2015-04-29.3 | MK546612 | MK546612 | — | — |
| *Homortomyces combreti* | CPC 19808 | NR_120215 | NG_059480 | — | — |
| *Homortomyces tamaricis* | MFLUCC 13-0441 | NR_155161 | NG_059495 | KU870905 | — |
| *Hysterium angustatum* | CBS 236.34 | — | FJ161180 | GU397359 | FJ161117 |
| *Hysterobrevium smilacis* | CBS 114601 | — | FJ161174 | FJ161135 | FJ161114 |
| *Hysteropatella clavispora* | CBS 247.34 | — | AY541493 | DQ678006 | DQ677955 |
| *Hysteropatella elliptica* | CBS 935.97 | — | DQ767657 | EF495114 | DQ767647 |
| *Inocyclus angularis* | VIC 39747 | NR_154069 | NG_060316 | NA | — |
| *Jahnula aquatica* | R68-1 | JN942354 | EF175655 | EF175633 | — |
| *Kellermania confusa* | CBS 131723 | NR_120192 | NG_042701 | NG_062176 | — |
| *Kellermania dasylirionicola* | CBS 131720 | NR_120194 | NG_042703 | NG_062177 | — |
| *Kellermania micranthae* | CBS 131724 | NR_120197 | NG_042706 | NG_062153 | — |
| *Kirschsteiniothelia phoenicis* | MFLUCC 18-0216 | NR_158532 | NG_064508 | MG859979 | MG994912 |
| *Kirschsteiniothelia rostrata* | MFLUCC 15-0619 | NR_156318 | NG_059790 | NG_063633 | — |
| *Kirschsteiniothelia tectonae* | MFLUCC 12-0050 | NR_148089 | KU764707.1 | — | — |
| *Lembosina aulographoides* | CPC 33049 | MN313809 | MN317290 | — | MN313839 |
| *Leptosphaeria doliolum* | CBS 505.75 | JF740205 | GU301827 | GU296159 | KY064035 |
| *Lichenoconium aeruginosum* | CBS 129239 | NR_160250 | NG_060266 | — | — |
| *Lichenoconium erodens* | CBS 128704 | MH864965 | MH876415 | — | — |
| *Macrophomina* *phaseolina* | CBS 227.33 | KF951627 | DQ377906 | — | — |
| *Melomastia italica* | MFLUCC 15-0160 | — | MG029458 | MG029459 | — |
| *Microthyrium buxicola* | MFLUCC 15-0213 | — | KT306552 | KT306550 | — |
| *Microthyrium propagulensis* | CBS 115976 | — | GU301846 | GU296175 | GU371734 |
| *Minutisphaera aspera* | DSM 29478 | NR_154621 | NG_060319 | NG_065059 | — |
| *Minutisphaera japonica* | KTC 2738 | AB733437 | AB733440 | AB733434 | — |
| *Murilentithecium clematidis* | MFLUCC 14-0562 | KM408757 | KM408759 | KM408761 | KM454447 |
| *Muripulchra aquatica* | KUMCC 15-0276 | KY320534 | KY320551 | — | MH551058 |
| *Muritestudina chiangraiensis* | MFLUCC 17-2551 | MG602247 | MG602248 | MG602249 | MG602250 |
| *Murramarangomyces corymbiae* | CPC 33000 | MG386045 | MG386098 | — | — |
| *Muyocopron atromaculans* | BPI GB1369 | NR_164053 | NG_066446 | — | MK492713 |
| *Muyocopron dipterocarpi* | MFLUCC 14-1103 | — | NG_059661 | NG_065647 | KY225779 |
| *Muyocopron laterale* | FMR13797 | MK874615 | MK874616 | — | MK875802 |
| *Muyocopron lithocarpi* | MFLUCC 14-1106 | MK347717 | KU726967 | KU726970 | KY225780 |
| *Myriangium hispanicum* | CBS 247.33 | KX887304 | GU301854 | GU296180 | KX887184 |
| *Myrmaecium fulvopruinatum* | CBS 139058 | NR_145390 | KP687861 | KP687968 | KP687936 |
| *Myrmaecium rubrum* | CBS 109505 | MH862829 | GU456324 | GU456303 | GU456344 |
| *Mytilinidion resinicola* | CBS 304.34 | NR_160068 | NG_057807 | NG_016511 | FJ161120 |
| *Mytilinidion scolecosporum* | CBS 305.34 | NR_160069 | NG_057808 | NG_016510 | — |
| *Natipusilla decorospora* | ILL S AF236 | — | NG_060263 | NG_061107 | — |
| *Natipusilla limonensis* | ILL S AF286 | — | NG_060264 | NG_062150 | — |
| *Natipusilla naponensis* | ILL S_AF217 | — | NG_060265 | NG_062151 | — |
| *Neodactylaria obpyriformis* | CBS 142668 | — | MK562751 | MK562750 | MK562752 |
| *Neodactylaria simaoensis* | YMF 1.3984 | MH379209 | MH379210 | MK562747 | MK562749 |
| *Oncopodiella trigonella* | FMR_10788 | KY853455 | KY853516 | KY853548 | — |
| *Parawiesneriomyces syzygii* | CPC 26528 | — | KX228339 | — | — |
| *Parmularia styracis* | VIC 42447 | NR_154315 | KP143728 | — | — |
| *Parmularia styracis* | VIC 42450 | KP273231 | KP143729 | — | — |
| *Parmularia styracis* | VIC 42587 | KP273232 | KP143730 | — | — |
| *Patellaria atrata* | CBS 958.97 | — | GU301855 | GU296181 | GU371726 |
| *Patellaria atrata* | SQUCC 15117 | MW077143 | MW077152 | — | MW276075 |
| *Patellaria quercus* | CPC 27232 | NR_152540 | NG_059696 | — | — |
| *Phaeoseptum terricola* | MFLUCC 10-0102 | MH105778 | MH105779 | MH105780 | MH105782 |
| *Phaeotrichum benjaminii* | CBS 541.72 | MH860561 | NG_057709 | NG_062645 | — |
| *Phyllobathelium anomalum* | MPN242 | — | GU327722 | JN887386 | MN562645 |
| *Phyllosticta ericarum* | CBS 132534 | NR_111759 | NG_042678 | — | — |
| *Phyllosticta maculata* | CPC 18347 | NR_147336 | NG_059472 | — | — |
| *Pirozynskiella laurisilvatica* | CBS 138109 | NR_153488 | NG_058462 | — | — |
| *Pleospora herbarum* | CBS 191.86 | KC584239 | DQ247804 | DQ247812 | KC584471 |
| *Polycoccum vermicularium* | Diederich 17545 | — | KT383808 | — | — |
| *Pseudoarthrographis phlogis* | CPC 32759 | NR_160349 | NG_064540 | — | — |
| *Pseudofusicoccum stromaticum* | CBS 117449 | — | DQ377932 | EU673147 | — |
| *Pseudogliophragma indicum* | MTCC:11985 | — | KM052851 | — | — |
| *Rhexothecium globosum* | CBS 955.73 | MH860827 | MH872561 | — | — |
| *Rhizodiscina lignyota* | G.M. 2016-10-12.No1631 | KY945339 | KY945339 | — | — |
| *Rhizodiscina lignyota* | G.M. 2017-01-12.1 | MF599193 | MF599193 | MF599193 | — |
| *Rhytidhysteron neorufulum* | MFLUCC 13-0216 | NR_164242 | NG_059649 | NG_063598 | — |
| *Rhytidhysteron thailandicum* | MFLUCC 14-0503 | NR_164241 | NG_059648 | NG_063597 | — |
| *Rupestriomyces ampulliform* | CGMCC 3.17061 | KF513536 | KF680799 | KF513507 | — |
| *Rupestriomyces sinensis* | CGMCC 3.17066 | KF513521 | KF680789 | KF513500 | — |
| *Rupestriomyces torulosus* | CGMCC 3.17051 | KF513527 | KF680791 | KF513505 | — |
| *Rupestriomyces torulosus* | CGMCC 3.17054 | KF513528 | KF680792 | KF680801 | — |
| *Saccharata intermedia* | CBS 125546 | NR_156539 | NG_057856 | — | KX464064 |
| *Schismatomma decolorans* | AFTOL-ID 307(=Ertz 5003 (BR) | AY548808 | AY548815 | AY548809 | DQ883715 |
| *Septorioides pini-thunbergii* | CBS 473.91 | NR_145234 | NG_058002 | — | — |
| *Spissiomyces* *aggregatus* | CGMCC 3.17072 | KF513518 | KF680779 | KF513498 | — |
| *Spissiomyces aggregatus* | CGMCC 3.17073 | KF513519 | KF680778 | KF680800 | — |
| *Spissiomyces* *endophytica* | SDBR- CMU319 | MF990800 | MF990799 | MF990798 | — |
| *Spissiomyces* *ramosus* | CGMCC 3.17075 | KF513516 | KF680785 | KF513496 | — |
| *Spissiomyces ramosus* | CGMCC 3.17077 | KF513515 | KF680783 | KF513495 | — |
| *Stigmatodiscus enigmaticus* | CBS 132036 | NR_164392 | KU234108 | NG_061223 | KU234121 |
| *Strigula jamesii* | MPN548 | — | JN887404 | JN887388 | — |
| *Sydowia* *polyspora* | CBS 116.29 | — | DQ678058 | DQ678005 | — |
| *Sympoventuria capensis* | CBS 120136 | NR_121323 | NG_057984 | NG_061163 | — |
| *Szaferohypha enigmatica* | G191 = CBS 152426 | PQ479987 | PQ479989 | PQ479988 | PQ475069 |
| *Trichodelitschia bisporula* | CBS 262.69 | MH859305 | GU348996 | GU296202 | GU371802 |
| *Trichodelitschia munkii* | Kruys 201 | — | DQ384096 | DQ384070 | — |
| *Trypethelium virens* | AFTOL-ID 1774 | — | KT232219 | — | — |
| *Tubeufia guangxiensis* | MFLUCC 17-0045 | MG012025 | MG012018 | — | MG012011 |
| *Tubeufia paludosa* | CBS 120503 | — | GU301877 | GU296203 | — |
| *Tumidispora shoreae* | MFLUCC 14-0574 | — | KT314074 | KT314076 | — |
| *Valsaria insitiva* | CBS 127882 | KP687886 | NG_064242.1 | KP687980 | KP687959 |
| *Valsaria spartii* | CBS 139070 | KP687843 | KP687843 | KP687964 | KP687919 |
| *Venturia catenospora* | CBS 447.91 | MH862259 | NG_064157 | — | — |
| *Wiesneriomyces conjunctosporus* | BCC4027 | — | KJ425449 | — | — |
| *Yuccamyces pilosus* | CBS:579.92 | MG386044 | MG386097 | — | — |
| *Zalaria obscura* | DAOM C 250849 | NR_153466 | KX579100 | NG_061249 | KX579106 |
| *Zeloasperisporium cliviae* | CBS 139915 | NR_138000 | NG_058173 | — | — |
| *Zeloasperisporium eucalyptorum* | CBS 124809 | NR_137732 | NG_057835 | — | — |
| *Zeloasperisporium pterocarpi* | MFLUCC 17-0910 | MH763754 | MH763755 | MH763756 | — |
| *Zeloasperisporium siamense* | IFRDCC 2194 | — | NG_059944 | JQ036223 | — |
| *Zwackhiomyces coepulonus* | RO31 | — | KU556870 | KU556969 | — |
